# Contact forces during hybrid atrial fibrillation ablation: an *in vitro* evaluation

**DOI:** 10.1007/s10840-015-0089-y

**Published:** 2016-01-04

**Authors:** Pieter W. J. Lozekoot, Monique M. J. de Jong, Sandro Gelsomino, Orlando Parise, Francesco Matteucci, Fabiana Lucà, N. Kumar, Jan Nijs, Jens Czapla, Paul Kwant, Daniele Bani, Gian Franco Gensini, Laurent Pison, Harry J. G. M. Crijns, Jos G. Maessen, Mark La Meir

**Affiliations:** Department of Cardiothoracic Surgery, Maastricht University Medical Center, Maastricht, The Netherlands; Department of Cardiothoracic Surgery, Universitair Ziekenhuis, Brussels, Belgium; Department of Pathology, University of Florence, Florence, Italy; Department of Cardiothoracic Surgery, University of Florence, Florence, Italy; Department of Cardiothoracic Surgery, Cardiovascular Research Institute Maastricht—CARIM, Universiteitssingel 50, 6229 ER Maastricht, The Netherlands

**Keywords:** Atrial fibrillation, Catheter ablation, Atrial arrhythmias, Computer model simulation, Contact force

## Abstract

**Purpose:**

Data on epicardial contact force efficacy in dual epicardial–endocardial atrial fibrillation ablation procedures are lacking. We present an *in vitro* study on the importance of epicardial and endocardial contact forces during this procedure.

**Methods:**

The *in vitro* setup consists of two separate chambers, mimicking the endocardial and epicardial sides of the heart. A circuit, including a pump and a heat exchanger, circulates porcine blood through the endocardial chamber. A septum, with a cut out, allows the placement of a magnetically fixed tissue holder, securing porcine atrial tissue, in the middle of both chambers. Two trocars provide access to the epicardium and endocardium. Force transducers mounted on both catheter holders allow real-time contact force monitoring, while a railing system allows controlled contact force adjustment. We histologically assessed different combinations of epi-endocardial radiofrequency ablation contact forces using porcine atria, evaluating the ablation’s diameters, area, and volume.

**Results:**

An epicardial ablation with forces of 100 or 300 g, followed by an endocardial ablation with a force of 20 g did not achieve transmurality. Increasing endocardial forces to 30 and 40 g combined with an epicardial force ranging from 100 to 300 and 500 g led to transmurality with significant increases in lesion’s diameters, area, and volumes.

**Conclusions:**

Increased endocardial contact forces led to larger ablation lesions regardless of standard epicardial pressure forces. In order to gain transmurality in a model of a combined epicardial–endocardial procedure, a minimal endocardial force of 30 g combined with an epicardial force of 100 g is necessary.

## Introduction

In patients with atrial fibrillation (AF), long-term restoration of sinus rhythm improves survival [1], enhances hemodynamics [2], and decreases the risk of thrombo-embolic events. Several invasive procedures are employed to restore normal sinus rhythm, but none has been proven to be fully successful. Percutaneous catheter ablation procedures are increasing in popularity [3, 4], but in order to be successful, multiple consecutive ablations might be necessary in one patient [5]. The “cut-and-sew” Cox-maze and the catheter-based Cox-maze IV are technically difficult but achieve higher single-procedure success rates [6, 7]. These invasive techniques have been mostly limited to patients who require concomitant cardiac surgery [8, 9]. Minimally invasive ablation procedures have been developed but show inconsistent results, especially in persistent and long-standing persistent AF [10]. Furthermore, their relatively high complication rate suggests that these techniques require further refinement [11].

A single-step hybrid ablation has been recently introduced [12, 13]. It combines in one procedure minimally invasive epicardial ablation with a percutaneous endocardial procedure in order to optimize lesion efficacy. The encouraging positive 1-year results reported using this technique might be explained by higher probabilities of transmurality obtained by ablating sequentially from the epicardium towards the endocardium and vice versa [14].

Recent improvements in catheter technology has given the EPs specialized catheters using contact force sensors located in the tip to optimize lesion efficacy and to avoid atrial perforations [15–20].

Epicardial ablation catheters are not equipped with such contact force sensors. Therefore, the optimization of transmurality by understanding the different contact forces employed might be important to better predict the lesion efficacy. We report an *in vitro* study assessing the efficacy of the different combinations of epicardial and endocardial ablation forces during radiofrequency (RF) hybrid ablation.

## Materials and methods

### ABLA-BOX design

**Fig. 1:**
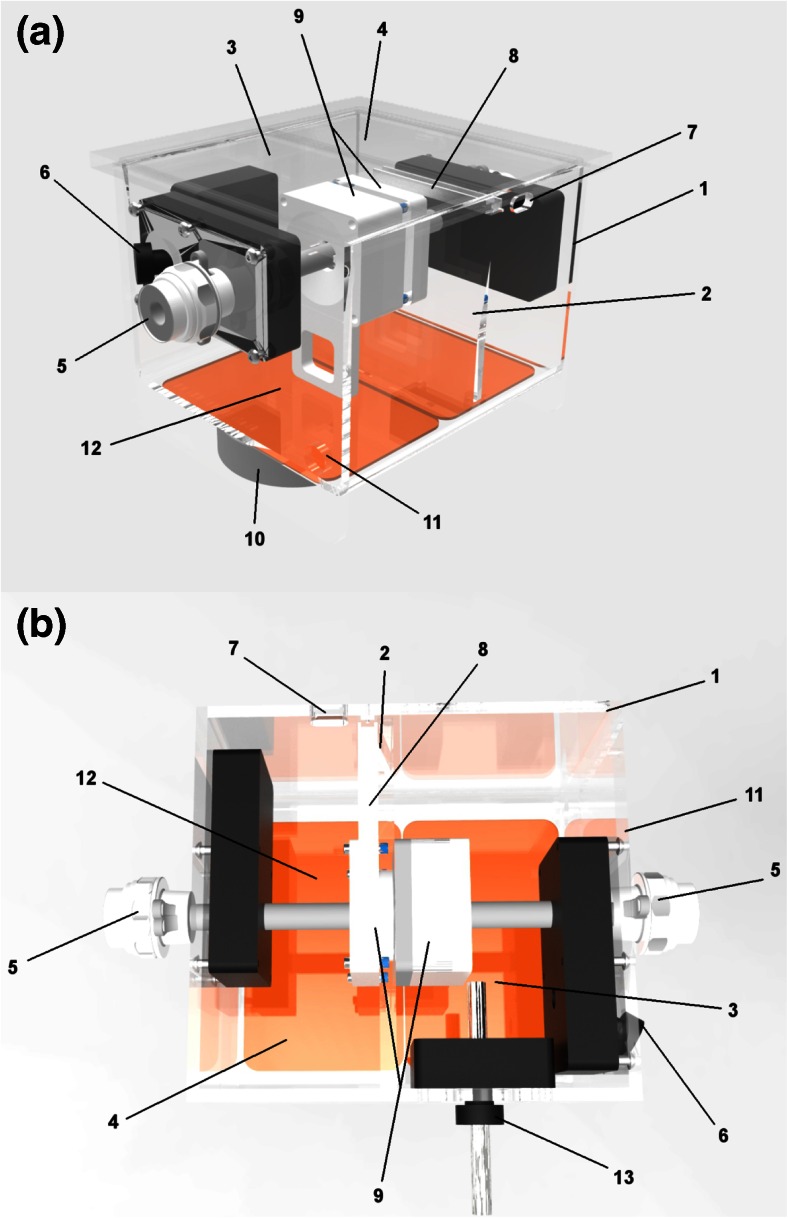
**a**–**b** The ABLA-BOX. *1* plexiglass box, *2* plexiglass septum, *3* endocardial compartment, *4* epicardial compartment, *5* 12-mm trocar, *6* linear lesion entrance, *7* thermocouple entrance, *8* rail for thermocouples, *9* magnetic tissue holder, *10* stirring motor, *11* outlet, *12* heating mat, *13* inlet

**Fig. 2:**
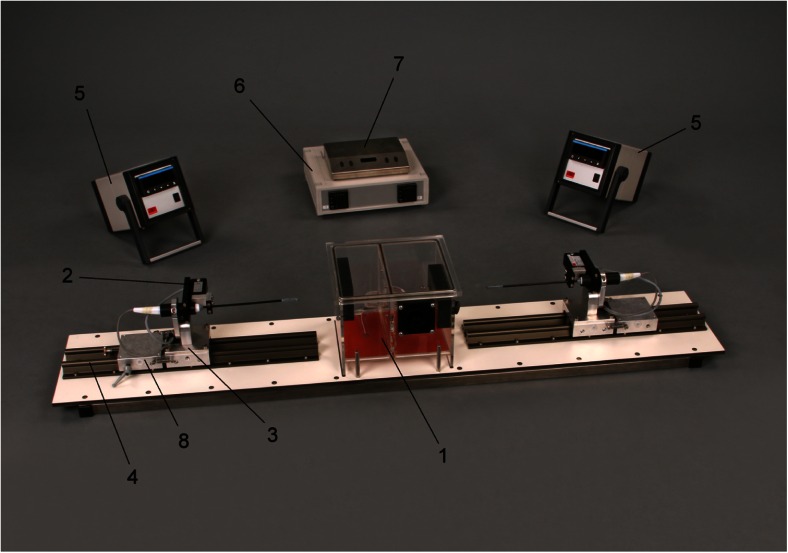
The ABLA-BOX setup. *1* plexiglass box, *2* force transducer, *3* catheter holder, *4* catheter holder rail, *5* digital forces monitor, *6* heating mat temperature controller, *7* stirring motor controller, *8* catheter holder fine-tuning system

The ABLA-BOX (IDEE—Instrument Development Engineering & Evaluation, Maastricht University, NL) is a plexiglass box construction (12.5 × 18.5 × 19.5 cm [*l* × *h* × *d*]) with two compartments separated by a plexiglass septum, mimicking the endocardial and epicardial cavities of the heart (Figs. 1 and 2). On a centered cut out, a magnetic tissue holder is placed, securing the specimen to the plexiglass septum, while allowing both endocardial as well as epicardial catheter access to the cardiac tissue. A heating mat (IDEE, Maastricht University, NL) ensures adjustable, selective, chamber temperatures for both endocardial and epicardial chambers. The epicardial chamber is filled with 1 cm of saline solution (NaCl 0.9 %). Together with the heating mattress, the vaporization of this fluid causes a humid environment, mimicking the epicardial space. Cather access is gained by two opposing 12-mm ports in line with the centered cutout of the septum. A special sealing system prevents endocardial leaking of blood. Both catheters are mounted onto an external railing system allowing controlled catheter positioning with a geared mechanism, while a force transducer (AE sensors, Dordrecht, NL), secured on the catheter holders, gives real-time feedback of contact forces during the ablation procedures (Fig. 2).

**Fig. 3:**
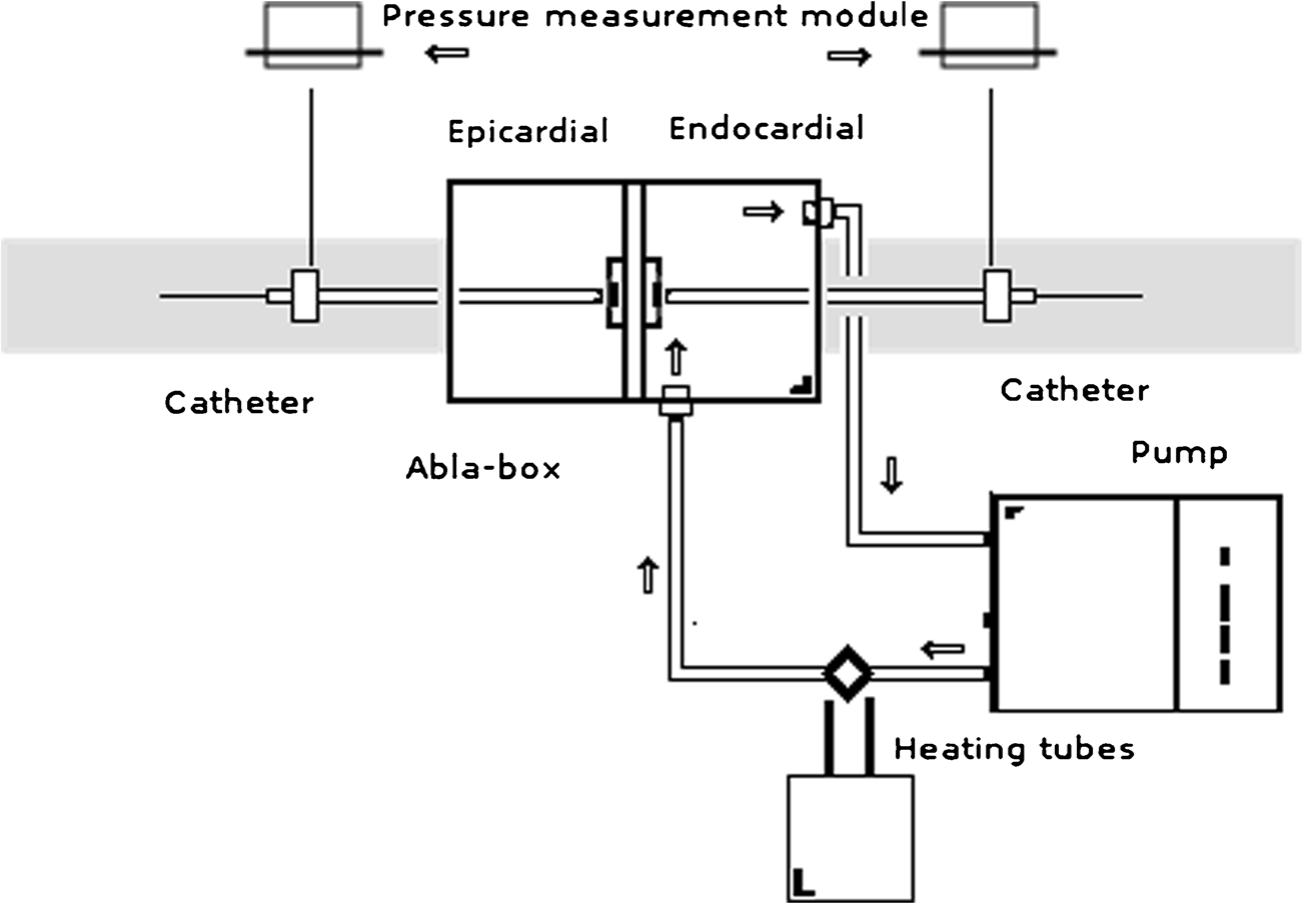
The *in vitro* circuit explained. A roller pump circulates blood through a circuit consisting of a heat exhanger and the ABLA-BOX

Cardiac blood flow is mimicked using an inlet and an outlet on the endocardial chamber, which are connected to an *in vitro* circuit. This circuit (Fig. 3) comprises of a pump (COBE precision blood pump, COBE Cardiovascular Inc., Arvada, CO, USA), a heating exchange device (Bio Cal 370, Medtronic, Minneapolis, MN, USA), and inlet and outlet 3/8 in. tubing (Maquet, Rastatt, DE). Three liters of freshly obtained and heparinized (25.000 U/L, LEO Pharma, Lier, BE) porcine blood fills the circuit, while the heating exchanger warms the blood returning to the oxygenator (set at 38.0 °C) and then to the endocardial chamber.

### Experimental setup

Left atrial tissue specimens were taken from freshly obtained pig hearts (posterior wall just above the mitral annulus). These samples (approximately 2.5 × 2.5 cm) were mounted in the tissue holder and magnetically fixed to the plexiglass septum with a central opening allowing catheter access on both sites of the tissue. An epicardial catheter (Isolator long-pen TT, Atricure, Cincinnati, OH, USA) was fixed on the epicardial catheter holder, while its endocardial counterpart was accommodated with an endocardial catheter (Biosense Webster, Inc., Diamond Bar, CA, USA).

Ablation was sequentially performed mimicking the hybrid approach: epicardial ablation was achieved first followed by the endocardial step. Epicardial contact force was set up at the chosen value, after which the catheter was secured on the railing system and ablation was performed. After the epicardial procedure, this catheter was removed; the endocardial contact force was regulated and fixed and endocardial ablation was performed.

Ablations were performed under similar conditions (blood flow 5 L/min, heat exchanger 38.0 °C, heating mat 37.5 °C) except from contact force. Each RF ablation was delivered at 15 W and was maintained for a maximum of 30 s unless an impedance rise occurred. When an impedance rise was observed, ablation was continued for another 10 s before ceasing ablation. Three different epicardial contact forces (100, 300, and 500 g) were paired with four distinct endocardial contact forces (20, 30, 40, 50 g). Each combination was evaluated five times, resulting in a total of 60 tissue samples.

### Morphometric evaluation of myocardial tissue ablation

Ablated left atrial tissue samples (±3 mm) were cut and fixated in formalin for at least 48 h. The samples were subsequently cryoprotected by immersion in a sucrose solution (20 %) for a period of 30 min and frozen at −20 °C in a cryostat (CM 1950, Leica Biosystems, Milan, Italy). The samples were placed on a flat support, to be cut in consecutive sections (100 μm), parallel to the endocardial surface. Obtained sections were collected in rows, on an acetate sheet and dried for 1 h at room temperature. At this point the ablated myocardial tissue area was clearly visible as a darker spot on a pale background. The maximum and minimum diameters and surface area of the ablated myocardium were measured by computer-aided morphometry (Image J v.1.48 software, National Institute of Health, Bethesda, MD, USA) after digitalization of the obtained sections.

The volume calculation of the ablated myocardium was achieved by multiplying the ablated surface area in each section by its thickness (assuming homogenous ablation through the whole ablated section) and superimposing the individual volumes of consecutive sections as a z-stack. For every lesion, the maximum diameter (*D*_max_), minimum diameter (*D*_min_), lesion’s area (*A*), and lesion’s volume (*V*) were reported by two individual observers, whereas the average measurement of these two was used for further analysis.

### Statistical analysis

Data were expressed as means ± standard deviations. Lesion measurements were analyzed using one-way ANOVA, after which post hoc testing was performed using the Bonferroni correction. A value of *p* < 0.05 was considered statistically significant. Intra-observer variability was tested on five samples randomly chosen and *κ*-statistics were used to determine the degree of intra-observer and inter-observer agreement after correction for the agreement expected by chance. A *κ*-value has a maximum of 1.0 when agreement is perfect. A value of 0 indicates no agreement better than chance agreement. All statistical analysis was conducted using SPSS v.18.0 (IBM Corp., Armonk, NY, USA).

## Results

The observers showed good agreement for *D*_max_ (*κ* = 0.88), for *D*_min_ (*κ* = 0.9), for *A* (*κ* = 0.86), and *V* (κ = 0.93). The mean *κ*-value was 0.89 ± 0.02.

**Fig. 4:**
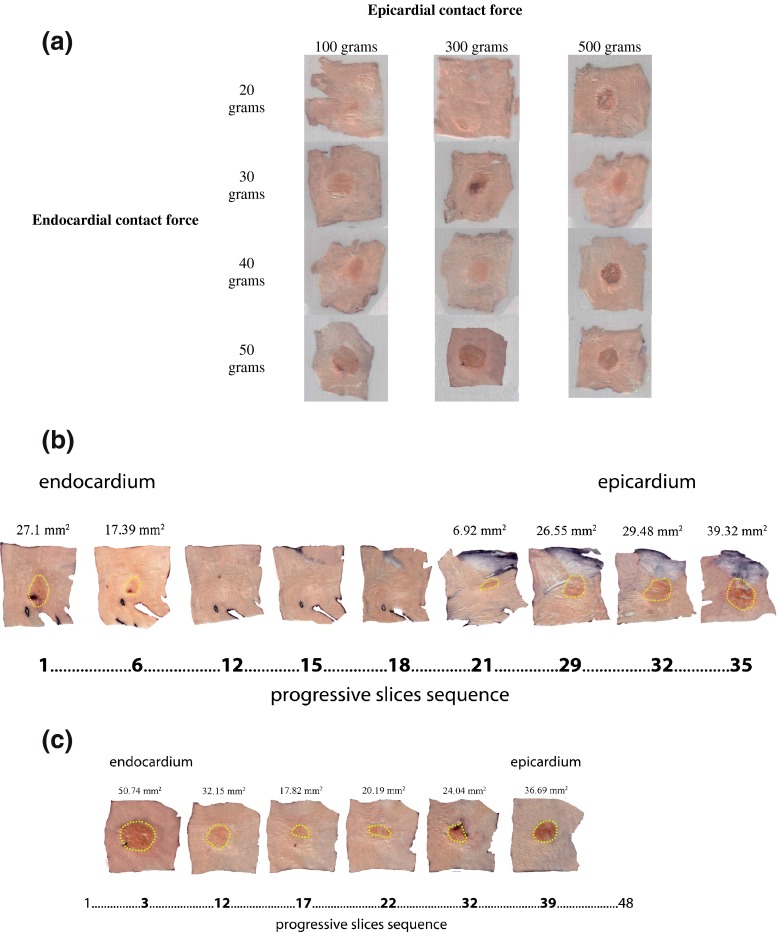
**a** Samples of middle sections of the left atrial tissue after *in vitro* hybrid ablation, employing different contact forces, cryopreserved and cut in consecutive sections. One hundred-micrometer thick, parallel to the endocardial surface. See text. **b** Ablation lesion formation progression during different tissue depths in a non-transmural ablation sample (endocardial force 20 g, epicardial 100 g), cryopreserved and cut in consecutive sections, 100-μm thick, parallel to the endocardial surface. **c** Ablation lesion formation progression during different tissue depths in a transmural ablation sample (endocardial force 30 g, epicardial 100 g), cryopreserved and cut in consecutive sections, 100-μm thick, parallel to the endocardial surface

With an epicardial contact force set at 100 and 300 g, no transmurality was obtained in combination with an endocardial contact force of 20 g (Table 1, Fig. 4). All other combinations tested in this study led to transmural lesions. The mean sample thickness varied between 3.9 and 5.0 mm overlooking the whole sample set (Table 2). The mean sample thickness did differ significantly when applying 20 g endocardially in combination with either 100 (3.9 ± 0.7 mm) or 300 g epicardially (4.8 ± 0.5 mm). Similar observations were found regarding the samples used for our research in the 40-g endocardial ablation groups: The mean sample thickness of the tissue ablated with 100 g epicardially was significantly larger (5.0 ± 0.5 mm) in comparison with the samples ablated with the combination of 500 g epicardially (4.3 ± 0.3 mm).

**Table 1:** Lesion dimension by contact force

			Epicardial CF		
			100 g	300 g	500 g
	20 g	Max diam. area (mm)			6.72 ± 2.15*
		Min diam. area (mm)	NT	NT	5.23 ± 1.82*
		Total area (mm^2^)			39.61 ± 23.25*
		Total volume (mm^3^)			3.96 ± 2.32*
Endocardial CF	30 g	Max diam. area (mm)	6.97 ± 1.51	6.72 ± 2.15*	7.85 ± 1.83*
		Min diam. area (mm)	5.42 ± 1.28	5.23 ± 1.82*	6.27 ± 1.60^†‡^
		Total area (mm^2^)	40.30 ± 16.20	39.61 ± 23.25*	53.44 ± 24.15^†‡^
		Total volume (mm^3^)	4.03 ± 1.62	3.96 ± 2.32*	5.34 ± 2.4^†‡^
	40 g	Max diam. area (mm)	7.29 ± 2.15	7.71 ± 1.96*	8.46 ± 1.93^†‡^
		Min diam. area (mm)	5.70 ± 1.89	6.07 ± 1.70*	6.73 ± 1.71^†‡^
		Total area (mm^2^)	46.26 ± 29.58	50.79 ± 24.18*	60.96 ± 27.18^†‡^
		Total volume (mm^3^)	4.63 ± 2.96	5.08 ± 2.42*	6.10 ± 2.72^†‡^
	50 g	Max diam. area (mm)	8.42 ± 2.29	8.91 ± 1.75*	9.46 ± 1.87*
		Min diam. area (mm)	6.70 ± 2.05	7.12 ± 1.56*	7.37 ± 1.68*
		Total area (mm^2^)	61.79 ± 35.36	66.90 ± 27.18*	71.68 ± 29.45*
		Total volume (mm^3^)	6.18 ± 3.54	6.69 ± 2.72*	7.17 ± 2.95*

*CF* contact force, *NT* not transmural

*No significant difference mean epicardial contact forces, ^†^significant difference means epicardial contact force 500 g vs. epicardial contact force 100 g, ^‡^significant difference means epicardial contact force 500 g vs. epicardial contact force 300 g; *p* ≤ 0.05

**Table 2:** Mean tissue thickness of the different used tissue samples

		Epicardial CF		
		100 g (mm)	300 g (mm)	500 g (mm)
Epicardial CF	20 g	3.9 ± 0.7	4.8 ± 0.5^§^	4.5 ± 0.2*
	30 g	4.3 ± 0.1	4.4 ± 0.1*	4.1 ± 0.3*
	40 g	5.0 ± 0.5	4.5 ± 0.4*	4.3 ± 0.3^†^
	50 g	4.3 ± 0.3	4.3 ± 0.2*	4.6 ± 0.1*

*CF* contact force

*No significant difference mean epicardial contact forces, ^†^significant difference means epicardial contact force 500 g vs. epicardial contact force 100 g, ^‡^significant difference means epicardial contact force 500 g vs. epicardial contact force 300 g, ^§^significant difference means epicardial contact force 300 g vs. epicardial contact force 100 g; *p* ≤ 0.05

Combining an endocardial force of 30 g together with an epicardial contact force of 100 or 300 g did not show any significant differences regarding the ablation’s maximum diameter area, minimal diameter area, total area, and total volume. Hence, combining the same endocardial contact force with 500 g led to a significant increase in all the parameters of the ablation lesion except for the maximum diameter.

Similar observations were made when combining an endocardial force of 40 g with different epicardial forces. Again, no significant changes were seen when this endocardial ablation was combined with 100 or 300 g, but increasing the epicardial contact force to 500 g led to a significant increase in the ablation lesion’s characteristics.

Combining an endocardial contact force of 50 g with increasing epicardial contact forces did not lead to significant changes in ablation lesion’s characteristics.

## Discussion

A combined epi-endocardial approach might provide an alternative to conventional catheter ablation therapy if this procedure is safe [13] and has favorable results [14]. Novel endocardial catheters have been developed that provide instantaneous feedback on the degree and orientation of force with which the catheter is contacting the atrial wall [15–19]. In contrast, although the development of new instruments and new technologies during the last few years has allowed complex epicardial procedures [21], poor attention has been paid to epicardial contact forces [22, 23]. In several studies, a strong relationship between endocardial contact forces and lesion depth was observed, being independent of delivered power [24–26]. Pressures lower than 10 g were associated with reduced success rates [27, 28]. Furthermore, it has been shown that the use of contact force catheters was associated with a significant improvement in freedom from antiarrhythmic drugs in comparison with standard catheters (88 vs. 66 %) albeit at the expenses of increased procedural and fluoroscopy times [29]. While insufficient contact force might result in ineffective lesions, excessive force might lead to a variety of complications including myocardial perforation, esophageal injury, and thrombus formation [25, 30, 31]. Based on *in vitro* research, it has been shown that the minimal force necessary to perforate atrial tissue can be as low as 38 g in RF-ablated sites [32]. This complication might be due to the structural weakening of the cardiac tissue due to collagen breakdown following high temperatures [33]. The use of contact force-guided ablation can lead to a decline in the occurrence of atrial wall perforations [20].

The importance of contact force could be even more significant for the dual epicardial–endocardial approach in determining the effectiveness of the procedure and guaranteeing patient safety. Indeed, by applying the energy to create myocardial scarring from both the endocardium and epicardium, the likelihood of tissue damage may increase, whereas an insufficient contact pressure may not result in full-thickness lesions, thereby abolishing the potential advantages of this new approach. Pison et al. published their initial experience with a thoracoscopic epicardial procedure combined with an endocardial AF ablation, a so-called single-step hybrid AF procedure [13]. A 30-mm-long irrigated bipolar radiofrequency probe was used to make the roof and inferior line connecting PVs from the right to the left side (box lesion). After epicardial ablation, an endocardial touch-up of these connecting lines was necessary in 23 % of patients because of gaps. The authors concluded that with currently available epicardial ablation tools on the beating heart, transmurality of the lesions cannot be guaranteed necessitating an endocardial touch-up [13].

There is no literature that has documented the relationship between applied forces and lesion’s size during an epicardial–endocardial atrial ablation procedure. Only a few studies have addressed the issue of contact force, using endovascular catheters, in epicardial ablations [22, 23]. On the left ventricular tissue with a predefined constant force ranging from 5 to 70 g, transmurality was not achieved, whereas on the thinner right ventricular tissue, transmurality was only obtained with a contact force of 70 g [23]. Clinically, combining conventional endocardial right ventricular tachycardia ablation with opposing epicardial ablation with 2–25 g led to favorable 6-month and 1-year results [22]. Wood et al. demonstrated that with a 30-mm-long irrigated bipolar radiofrequency probe, a 450 g contact pressure gave an epicardial lesion length of 31.3 mm and an endocardial lesion length of 14.1 mm. The average lesion depth was 4.2 ± 0.74 mm. Endocardial blood flow did not influence lesion depth. With 900 g contact pressure, increased depth was achieved with transmurality at 4.8-mm tissue thickness or less. The authors concluded that lesion depth was increased by greater pressure on the probe and was not affected by blood flow. Endocardial lesions were smaller than epicardial dimensions [34].

To the best of our knowledge, to date there are no epicardial catheters commercially available, which provide similar contact force-guided ablation options as seen in endocardial ablation. Epicardial ablation requires different angulations of approach, leading to profound contact force variation and positional “drifting”, which are associated with lesser impedance and reduced efficacy of lesion formation [35]. In accordance with one of our surgeons, known for his expertise in atrial fibrillation ablation surgery (M. L. M.), we measured the pressure he applies during a test run in our *in vitro* setup. This came out to be around 300 g so we made the assumption that a 100-g lower margin and 500-g upper margin seems feasible. Although these pressures are of a magnitude of order greater than those seen in endocardial application, we would like to accentuate that these catheters are hold in hand during such procedure, while working on a beating heart. Therefore, the application of forces on the epicardial side in the order of magnitude of 20–30 g is currently not feasible by humane application, which should be taken in consideration.

We performed an *in vitro* ablation study mimicking the hybrid approach, assessing the lesion size and volumes obtained from different combinations of conventionally used endocardial ablation contact forces, with three distinct epicardial contact forces. Our *in vitro* setup, the ABLA-BOX, allowed standardization of blood flow, circulating blood temperature, and both endo- and epicardial contact forces.

Our study has shown that the combination of an endocardial force of 30 g with 100 g applied epicardially led to scar transmurality. In contrast, the combination of a similar epicardial force with the minimally accepted endocardial contact force of 20 g (commonly used in endocardial ablations) resulted to be insufficient. Our findings also demonstrated that a combination of forces of 500 g epicardially, together with 20 g endocardially, also results in transmural scarring. The necessity of an endocardial ablation with limited epicardial forces (450 g) are in concordance with the results of Woods et al. [34]. We also observed that with increasing endocardial pressures and constant epicardial forces, a rise in maximum and minimum diameters, total area, and total volume can be seen. When applying an endocardial contact force of 50 g (upper safety limit for endocardial pressures), no significant changes occur in maximum and minimum diameters, total ablated area, and ablation volume. We might postulate that the combined epicardial–endocardial ablations could play a significant role in the final determination of ablation success by addressing the specific challenges related to each specific approach. The impact of epicardial RF lesions at areas with epicardial fat may be less effective since these lesions might be penetrating less deeply into the tissue covered by epicardial fat [23]. It can be also hypothesized that the blood flow on the endocardial side might play an important role in the heat transfer during ablation, thereby limiting the heat transfer to the tissue.

On the basis of our results, since sufficient transmurality was gained by using 30 g of endocardial force combined with the minimal epicardial contact force of 100 g, new catheters could be designed based upon existing endocardial technologies to achieve safe and effective ablation procedures in a combined epicardial and endocardial approach. However, further studies are necessary in order to translate our results into clinical practice.

### Study limitations

Although our findings provide useful insights in the physical background of the hybrid approach, the results obtained cannot be fully extrapolated to *in vivo* research due to some limitations which must be pointed out.

First, in our setup, we evaluated contact force perpendicular on a non-beating piece of myocardium, while clinical practice requires the creation of different ablation lines using different angulations of approach. Therefore, real-life catheter contact forces may vary significantly during ablation leading to profound contact force variation and “positional” drifting, associated with a lesser impedance and reduced efficacy [35]. If endocardial ablation had been performed in a more parallel catheter configuration, we hypothesize that the minimal endocardial force necessary could possibly have been similar to that found in other studies (10 g), due to improved catheter–tissue contact [27, 28].

In the current clinical practice, the effect of the ablation procedure is evaluated using endocardial mapping catheters in order to assess electrophysiological isolation. In our study, we histologically evaluated the lesions’ size and volume overlooking electrophysiological properties. This aspect must be considered when examining our data. During *in vivo* ablation procedures, permanent thermal injury occurs when temperatures reach 50 °C and above [36, 37], while temperatures above 80 °C are associated with increased thrombus formation [38]. These arguments have led to the development of the so-called irrigation-cooled ablation catheters [39, 40]. However, it has been shown that tissue temperatures may far exceed catheter tip temperatures. Once these temperatures exceed 100 °C, steam explosions can occur, which might be hearable as so-called “steam pops” [38, 41]. These small “explosions” may cause serious problems varying from superficial craters to deep tissue tears with the potential of developing in cardiac perforation or cardiac tamponade [42, 43]. In our current study, we have not monitored the periprocedural tissue temperatures, nor did we count the incidence of “steam pops”. This will be the object of ongoing studies.

Although the samples studied did show differences regarding mean sample thickness in two combinations of contact forces, we currently did not do any further analysis regarding these minor differences (<1.0 mm) because of its debatable clinical significance. Furthermore, the lack of trabeculation in our tissue samples will also contribute to a different heat exchange, when extrapolating our data to different parts of the atrium. Therefore, we postulate that more trabeculation leads to an increased heat exchange surface, leading to less effective ablation lesions. These two tissue-related issues remain subject for future research.

Lastly, the reader should keep in mind that although the results in our study are obtained in an *in vitro* model of AF ablation, standardizing a variety of parameters (e.g., blood flow, angulation of approach, etc.), there is no real tissue perfusion during the course of the experiment and heat dissipation should thus be regarded as artificial.

## Conclusions

Increased endocardial contact forces led to larger ablation lesions, while maintaining epicardial forces. In order to gain histological transmurality, an epicardial force of 100 g is sufficient when applying an endocardial force of 30 g. However, when performing epicardial ablation on the beating heart in patients, the necessary minimal contact forces could be significantly higher. Further research is warranted to confirm our findings.
